# The changing role of operational meteorology towards a transdisciplinary approach to future weather and climate services

**DOI:** 10.1098/rsta.2024.0535

**Published:** 2025-07-31

**Authors:** Paul Davies, Hayley J. Fowler, Helen Roberts, Christopher J. White, Monica Youngman, David P. Rogers

**Affiliations:** ^1^ Services, Met Office, Exeter, Devon, UK; ^2^ School of Engineering and Tyndall Centre for Climate Change Research, Newcastle University, Newcastle upon Tyne, UK; ^3^ Department of Civil and Environmental Engineering, University of Strathclyde, Glasgow, UK; ^4^ Office of Science and Technology Integration, National Weather Service, Asheville, NC, USA; ^5^ Global Facility for Disaster Reduction and Recovery, The World Bank, Washington DC, USA

**Keywords:** operational meteorology, transdisciplinary, weather and climate services, living labs

## Abstract

This paper describes the role of operational meteorology from a single disciplinary standpoint through to multidisciplinary, interdisciplinary and, more recently, transdisciplinary approaches and what this means for future forecasting systems. We start from the foundation of a single meteorological discipline: in this case, the forecasting of wind speed and direction that led to the creation of the first gale warning service in the wake of the ‘Royal Charter Storm’ in 1859. We build on this, giving further examples of where multidisciplinary and interdisciplinary methods have enhanced our understanding of observed meteorological and hydrometeorological phenomena from multiple perspectives, to produce a more holistic understanding of the effect of weather and climate on the ‘whole of society’. An exemplar of this is the integration of meteorology and hydrology to create the UK’s first hydrometeorological profession through the Flood Forecasting partnership between the Met Office and the Environment Agency. We end our discussion by exploring the move to a transdisciplinary approach through living labs: we believe this is vital to increase societal resilience to ever-more extreme weather events driven by anthropogenic warming.

This article is part of the Royal Society Science+ meeting issue ‘Hydrology in the 21st century: challenges in science, to policy and practice’.

## 1. Start of the journey—the beginnings of operational meteorology and disciplinary expertise and advice

Admiral FitzRoy established the Meteorological Office in the UK in 1854 [1]. As a user of weather data from his time in the Navy, FitzRoy was interested in using weather data to have a beneficial effect for society. FitzRoy was motivated to see if there was something his new department could do to help protect shipping from future disasters following the Royal Charter Storm in 1859 where 133 ships and 800 lives were lost.^1^ This included over 450 lives from the Royal Charter, which was driven ashore on to the east coast of Anglesey by Force 12 Beaufort scale winds. On 1 September 1860, weather reports began to be collected at the Meteorological Office in London via the electric telegraph and on 5 February 1861, the first storm warning was issued.[2] This service had all the elements that have characterized our weather forecasting services ever since: a focus on users’ requirements and needs, and an innovative approach to solving a problem. This was a ground-breaking service, exploiting new technology—the telegraph—without which, the collection of observations and dissemination of timely warnings would not have been possible. An additional element was the exploitation of our scientific understanding. What was learnt from FitzRoy’s analysis of the Royal Charter Storm made forecasting future storms from today’s observations a possibility. FitzRoy’s maps of the event would be very familiar to operational meteorologists today. In many ways, FitzRoy, with his focus on using knowledge and data to have a positive effect for society, could be thought of as the first operational meteorologist.

## 2. The key element of trust: from a single discipline origin

The science of meteorology continued to advance through the first half of the twentieth century, with accurate weather forecasting playing a vital role in World War II. This is personified in Group Captain Stagg,^2^ who led the meteorological advice in the lead up to D-Day and was responsible for arguably the most important—and effective—decision ever made by an operational meteorologist. The story is told that Stagg correctly forecast the deterioration of conditions ahead of the original scheduled D-Day landings of 5 June and the ‘weather window’ that allowed the invasion to go ahead on 6 June. Fortunately, Eisenhower and his senior military staff took their decisions based entirely on Stagg’s advice. When he warned of deterioration on the evening of the 2 June 1944, the sun was streaming through the window at the end of a beautiful day. Equally, on the evening of 4 June when he provided greater confidence in the forecast of a temporary clearance in the weather, rain was lashing against the window. Eisenhower’s team had differing views. Montgomery wanted to go whatever, while Air Marshall Tedder was reluctant to go without perfect flying conditions. Faced with conflicting views from his senior commanders, on both calls it came to Eisenhower to take the decision. And on both occasions, he backed his meteorologist, despite the weather outside the window being completely different to what was being forecast 24 h hence. Crucially, Stagg had Eisenhower’s trust. Eisenhower knew how important the weather was to the success of the invasion and so he had tried to develop a close working relationship with Stagg in the month before D-Day so that he could see how accurate his predictions were and how much he could trust him. And Stagg, similarly, completely understood the implications of his forecast and what was needed in terms of appropriate weather conditions to be able to launch a successful invasion. Mutual trust and the understanding of users’ needs was the starting basis of operational forecasting which continues to characterize the excellence in forecasting from operational meteorologists today.

## 3. Learning from the great storm of 1987: how do we enable action on the ground that saves lives and livelihoods? The role of multi-disciplinary investments

Despite all these impressive developments, in October 1987 the Met Office missed a storm which caused significant damage to the south of England [3]. There are two take-aways from the ‘Great Storm’ of 1987. First, despite access to satellite imagery which showed development of the storm, we were still learning what those images meant for the speed and strength of development, and the existence of features that would lead to harmful surface weather, such as a sting jet [4]. With new observations, operational meteorologists had to develop new conceptual models to understand what this meant [5]. Similarly, we were still learning the characteristics and fallibilities of numerical weather prediction (NWP) [6,7]. New exceptional technology requires time for excellent people to learn how to use it and apply it to provide extraordinary effect. A bit like the Royal Charter Storm over a hundred years previously, operational meteorologists used an event to increase knowledge and understanding of meteorology to be able to provide better forecasts in the future. By understanding how we could use the new source of knowledge—satellite observations—we could validate and challenge what computer models are showing.

The second takeaway from the Great Storm is that even if we had forecast it correctly there was no mechanism to warn anybody to act, meaning that a perfect forecast would have limited value [7]. Also, meteorologists’ more limited knowledge, at the time, of the ‘multi-hazard’ dimensions of the Great Storm, such as the antecedent conditions that weakened the environmental response to strong winds, and the compounding nature of other hazards that resulted in greater loss of life (heavy rain, landslides and coastal surges), meant that the storm caused greater effects than were anticipated.

Today, we are increasingly exposed to the perils of anthropogenically enhanced extremes and these multi-hazard scenarios. The effects of storm events are often underestimated due to the complex risks posed by the interconnections between hazards [8]. These complex interconnections are referred to as multi-hazard (or ‘compound’) events, where hazardous events may occur alone or simultaneously, be spatially or temporally compounding, and produce cascading or cumulative effects [9]. It is increasingly recognized that if two disasters occur simultaneously or in succession in the same place, the combination can be more effective than if they occur in isolation, and responders who could handle the effects of one event may potentially find themselves overwhelmed by successive events [10]. A recent example is provided by the effects of storm Bert in the UK, with strong winds and heavy rain leading to flooding and landslides. This storm was closely followed by storm Conall, which probably would have resulted in fewer effects had the ground not already been saturated by Bert [11]. It is also necessary to consider sectoral and infrastructure interdependencies, which span all natural hazardous risks.^3^ Resilience in one sector is highly dependent on resilience in another and therefore attention is needed to ensure that vulnerabilities in one sector do not compromise another [12].

Thus, what we must always bear in mind is that however good a forecast is, it is only as good as its use and application. And so, the other similarity to the Royal Charter Storm is that while that led to the development of the gale warning service, the 1987 Storm led to the development of the National Severe Weather Warning Service [13] that encompasses multiple hazards, not just wind-related hazards.

## 4. The Summer 2007 floods and impact-based forecasting and warning services: how interdisciplinary initiatives took hold

The progress with NWP since the Great Storm has been remarkable. A 4-day forecast is now as accurate as a 1-day forecast was 30 years ago [14]. This has been driven by: improvements in computing, which has enabled us to run NWP models at much higher resolution; better observing networks, especially satellites, which means our NWP models have a better representation of the actual state of the atmosphere and reduce errors in prediction; and, not least, better science, which has produced more sophisticated representations of the physics and dynamics of the atmosphere and ocean in our models.

Operational meteorologists, however, remain a vital part of our value chain. NWP models do not produce perfect forecasts and the rapidly changing climate, such as increasing sea surface temperatures, could be an issue for NWP accuracy in the future. Forecasts on the second to fifth day lead time are as good as they need to be for most purposes. But users are not just interested in forecasts—they want to know and understand the potential effects of the forecast. It is not just what the weather will *be* but what the weather will *do,* which can be provided through actionable impact-based forecasts (e.g. [15–18]) bridging to consequence forecasting [19].

The wet summer of 2007, which resulted in extensive flooding across the UK and almost catastrophic risks to some elements of national infrastructure, exposed a major problem with flood forecasting in England and Wales [20]. The Met Office forecast the rain, and the Environment Agency issued the flood warnings, but there was no effective working relationship between the two agencies at the time. It did not matter how well the rain was forecast; if the forecast was not being put to effective use in driving an accurate river model, a flood warning could not be issued. This led to the Pitt Review,^4^ an independent appraisal of flood risk management in England, which resulted in the creation of the Flood Forecasting Centre (a partnership between the Met Office and the Environment Agency) and, critically, the creation of a new breed of expert in the UK, an interdisciplinary profession: the hydrometeorologist. This brought together two disciplines to ensure that the effects of rainfall—flooding—were being translated into actionable warnings.

At the same time, we were evolving and developing the role of the civil contingency advisors, individuals working closely with responder organizations across the country to ensure that messaging around extreme weather warnings was getting to people that matter. Working with that community, we developed the first impact-based weather warning system in the world [13,21]. An important part of mitigating effects is our communication of warnings to the public. Evidence shows that giving the storm a name helps in the communication of severity [22], and in 2015 the Met Office, working with our Irish counterparts Met Eireann, launched a storm-naming system [23]. This has since taken off in partnership with colleagues across Europe. In addition, as of December 2023, national severe weather warnings include behavioural science informed advice that is specific to the hazard, effect and likelihood. These collective advice and decision-support offerings are not just based on the Met Office’s forecasting capabilities, but those of others too. Operational meteorologists now have a more vital role in providing independent advice than ever before. In effect what operational meteorologists are doing today—providing advice to people making critical operational decisions—does not sound too much different to what Stagg was doing in 1944.

## 5. What about the future? The role of ensembles, machine learning, seamless storylines and transdisciplinary collaboration through living labs and professional development

Human-induced climate change is causing more frequent and intense extreme weather events, such as extreme heat or severe thunderstorms, and making them more difficult to forecast (e.g. [24–26]). The concept of weather and climate as chaotic systems has had a profound effect on the way in which weather forecasting has evolved over recent decades. No longer do we produce a single, deterministic forecast, but instead we perform an ensemble of forecasts that seek to capture the plausible range of future states [27]. This provides a range of forecast solutions, which allows operational meteorologists to assess possible outcomes, estimate the risks and probabilities of those outcomes, and to gauge the level of confidence in the final forecast. From the users’ perspective, providing the information is presented in ways that are easy to understand [28], these forecast probabilities allow them to decide on the level of risk they are prepared to take depending on their vulnerabilities, and to take appropriate action within a proper understanding of the uncertainties [29].

However, key questions that are emerging are whether artificial intelligence (AI) methods could replace (non-AI) physics-based weather forecasting methods, and will AI change the role of the operational meteorologist [30]? In terms of replicating physics-based NWP, a fundamental question is whether the training dataset for the AI algorithm contains sufficient information to enable the predictions to be both accurate enough and sufficiently dynamically and physically realistic [31]. There are scientifically plausible arguments to suggest the information content in the currently available training datasets is far from sufficient. However, by some measures of the prediction skill, the prototype AI predictions appear competitive with those from (non-AI) physics-based NWP, which might suggest the information content is sufficient.

Furthermore, could AI augment, replicate, or even improve on the complex human decision-making processes during extreme events and thereby enable more efficient and effective mitigation actions to take place? There is increasing evidence that AI deep-learning software tools can generate weather forecasts much more rapidly than existing physics-based models; this would represent a significant benefit to the users of weather forecasts, but it remains unclear if they can be optimized to deliver good advice during acute life-threatening situations. The next important step in our journey will be to assess the physical and dynamical realism of forecasts as a measure of their quality, answering the question of ‘when do we know a weather forecast is good enough?’ Important aspects of this will be how good AI, human and physics-based approaches are in producing a good forecast and how do we measure the things that matter most to users so that we better understand the value of our services and where to improve.

We believe that the process of causal inference frameworks [32] could be used to combine observational causal inference and physical modelling. This process provides a complementary approach to commonly adopted ‘aleatory-based’ correlation, or statistically based techniques e.g. by putting emphasis on plausibility rather than probability. At its heart, a causal inference framework embraces data-driven causal methods through the process of pattern recognition and machine learning (ML), replicating in part the cognitive processes of an operational meteorologist. The development of these types of tools requires multidisciplinary expertise, including data science, physical science, behavioural and decision-science. It is arguable, and possibly unknown at present, whether this type of decision tool will replicate the operational meteorologist, or whether human meteorologists will still be required for quality control, and as advisors and consultants for customers.

Applying this new approach beyond that of discovering new science, to operational meteorology across all timescales from weather to climate (next frontier), could provide a new working environment, tools and processes for operational meteorology. This extended-range timescale—once regarded as a ‘predictability desert’ [33]—has seen recent skilful predictability advances on timescales up to 46 days ahead that have spurred an increasing interest in predicting extreme events [34] and producing improved early warning and actions [35]. Moreover, the creation of event-based storylines [36] can provide a way of combining lines of evidence from different observations and climate models (km-scale to global climate models) to develop causal pathways for projecting future ‘events’ [37]. We believe that using a causal inference framework will first deliver new science to improve weather forecasting methods, with a focus on improving meso-scale features, starting from our new conceptual model for extreme convective downpours [38], to improve hydroclimate extreme predictions. This links directly to common practices in disaster risk management using ‘stress-testing’ for emergency preparedness based on events that are conditional on specific and plausible assumptions. Since these event-based storylines allow for conditional explanations, without full attribution of every causal factor, crucial when some aspects of the latter are complex and highly uncertain [39], they also provide a natural route into thinking about changes to future events.

However, as described in the case of the Great Storm, improving predictions and forecasts will only provide value to the extent that they are used to promote effective action. Users must be able to receive, understand and act on the information provided. Increasing community resilience in advance of events requires decision-makers to combine complex, interdependent data, including infrastructure assets, utilities, communication networks, and socioeconomic data—with projections of (near or far) future weather effects. With extreme weather events increasingly being outside of the experience of people and communities, it is difficult to fully comprehend the level and scale of potential effects, in part due to psychological distance [40]. As our data and technology scale into digital twins to capture these interconnected systems, we will have the opportunity to not only predict local, specific effects for events, but to allow people to virtually experience these potential extremes using virtual reality (VR). It is very different to hear that a river will rise to 8 m than to see in three dimensions using augmented reality (AR) visualizations [41] how local businesses will be flooded to the rooftops and communities destroyed. There are also advantages for individual protective action decisions, with a review demonstrating that VR/AR can be advantageous compared with text and photographs in pre-disaster preparedness and hazard recognition and prevention due to the realistic and engaging visualizations [42], while Alshowair *et al*. [43] find VR better than tabletop exercises for disaster preparedness. In addition, city-scale digital twins combined with weather and climate information could be used to model how adaptation measures, whether a larger storm drain, a levee or additional shelter, would change the outcome. This could aid local decision-makers in highlighting risks and inform trade-offs in designing infrastructure improvements, community investments and zoning decisions as well as informing updating of building codes. As the telegraph enabled the initial weather warnings, today’s data and technology must be employed to revolutionize how we communicate these impact-based early warnings to ensure comprehension and facilitate action.

Our vision highlights the increasingly important role of social sciences [44]. Helping people and society to make better decisions requires understanding of human judgement and decision-making, and encouraging appropriate action in anticipation of, and preparation for, harmful weather must be informed by behavioural research and communication expertise. The role of a socio-meteorologist involves understanding the heuristics and cognitive biases we are all prone to as humans, that may affect our decisions. Knowing that providing more options leads to harder decision-making [45] means that mitigative advice in warnings should be limited in number. Yet, evidence suggesting that people rely on information that is recent or readily available, availability heuristic [46], means we must ensure that information is salient, perhaps visual, with comparison made to previous similar events; again, this naturally leads to a narrative or storyline approach. This becomes especially challenging for unprecedented extremes where people have no prior experience on which to base their decisions to act. Providing real-time, community-based, actionable warnings that include context on the current situation and are integrated with the local evolving situation, such as identifying available shelters or egress routes, would save lives. Socio-meteorology also pertains to the decision processes of the meteorologists, hydrometeorologists and civil contingency advisors who work collaboratively on decisions when issuing weather warnings, and may also be prone to cognitive biases, but whose judgements are critical for ‘safety of life’ messaging. These complex and multifaceted decisions must be robust and evidenced, to provide the greatest value to society.

The social sciences are also equally important in the age of climate science translators (CSTs) [47]. A new generation of professions that specialize in the brokering, translation and tailoring of climate science data to decision-makers is currently evolving, both independently and affiliated with humanitarian aid organizations, as well as within the business sector, to cope with the growing urgency and complexity of climate-related issues. Furthermore, there are many examples of the creation of living labs, which enable transdisciplinary and user-centric co-creation of value and services to benefit the economy, society and the environment [48–50]. Living labs, as physical or virtual spaces, bring together various stakeholders in which to solve societal challenges and this collective ideation would complement the professional development of CSTs as a viable future vision. This vision embraces transdisciplinary collaborations with social science and innovative, hybrid ML capabilities through living labs which together enable vastly improved services across weather and climate timescales so that the whole of society can thrive and be better prepared for the unexpected, unprecedented surprises and worst-case scenarios still to come.^5^

When faced with rapidly amplifying climate change and associated weather extremes, it is intriguing that the role of business and commerce in protecting lives and livelihoods does not attract higher importance. After all, these enterprises are focal points in many communities and are intricately linked to various aspects of the economy, environment and social fabric. Climate change poses significant risks to businesses, ranging from supply chain disruptions and increased operational costs, to reduced demand for goods and services [51,52] as well as duty of care for their employees. Therefore, an improved understanding of the complex interactions between different types of risk, and how these can compound, cascade and affect cross-trade-supply boundaries is vital. Moreover, the role of businesses in ensuring prosperity and community resilience is not always related to life-threatening scenarios. For many businesses, an unexpected period of ‘bad’ weather could have a significant effect, potentially comparable to the fallout following a spell of extreme weather [53].

Yet, often the subtle differences in weather variables, such as temperature and rainfall, may be too nuanced for most business managers. Announcing that there is a 30% chance of extreme rainfall and associated flash flooding without context or explanation on what actions to take is of little value to most people [54]. Therefore, whether a timely warning is issued is less important than whether everybody—including the most vulnerable, under-represented and disadvantaged in society—is taking the appropriate action. Businesses and communities can play an important role here. Knowing who is the most vulnerable and how best to message and advise those who need it most is the key. This is why increased inputs from social and behavioural sciences along with greater and more meaningful engagement with businesses and community organizations could help to avoid poor decision-making and address the lack of awareness of all types of threats affecting communities.

## 6. Conclusion

This paper describes the past and future role of operational meteorology from a single disciplinary standpoint through to multidisciplinary, interdisciplinary and recent transdisciplinary approaches. This is summarized with examples listed in table 1. There is no doubt that the role of the operational meteorologist will continue to evolve into the future as it has done so in the past. There will be a need to embrace new tools and skills to exploit the continuing investments in science, observations and technology that are improving our forecasts to meet the ever-growing demands of our users for greater accuracy and more tailored advice set against the backdrop of a changing climate. However, a sustainable collaboration between meteorologists, partners, and users involved in judgement and decision-making research will be necessary if the goal of improving effective decision-making and enabling extraordinary outcomes are to be achieved. As we start to consider new data and ML-enabled analytic tools and services, there is an urgent need to create and fund transdisciplinary thinking [55,56].

**Table 1.** An illustration of the relevant ‘disciplinary’ related services, underpinning science and application development activities and professions. The examples from the UK are not exhaustive and only used as a guide.

	disciplinary	multi-disciplinary	inter-disciplinary	trans-disciplinary
examples	single hazard	multi-hazard	impact-based	community-based
services (UK)	wind, heat, rainfall, flood warnings	natural hazards partnership assessments	flood forecasting centre flood guidance	seamless climate- weather adaptation and resilience services. Event-based storylines. ML/AI & social sciences enabled services
underpinning science and application development	ensemble-based first guess warnings	impact-based modelling (hazard, exposure and vulnerability)	earth system modelling	seamless, community-based modelling. Complex, compound, cascade and connected regime & event-based modelling. Coupled social, behavioural emulator simulators
profession	meteorologist, climatologist, hydrologist	geologist, volcanologist	hydrometeorologist, hydroclimatologist	CST, Socio-meteorologist, ML/AI engineers

In many cases, the extraordinary effect of operational forecasts and warnings comes down to a single piece of advice or guidance from a meteorologist who has a strong subject knowledge, having the trust of, and a deep understanding of the users, with the ability to convey complex information in a way which enables the user to make an effective decision. This is not so different from what Group Captain Stagg was doing in 1944. However, there is also the need to embrace a different way of working, to become more agile and to flex quickly to changing user demands. This agility is at the heart of transdisciplinary collaboration; one that will broaden the role of the operational meteorologist beyond that of the existing production treadmill, with greater emphasis on developing skills that users can trust and rely on, for example towards becoming a future CST. These roles will be increasingly important as climate extremes and the resulting effects become more severe. These attributes are not too different to that of a doctor, having the expertise to diagnose an unwell patient while having the skills to be empathetic, reliable and credible—all related to trust: a key ingredient in delivering value and benefits to the whole of society.

A ‘whole of society’ approach based on inclusivity and participation is needed to build resilience to extreme weather and climate change. This is immensely challenging. It requires collaboration among communities, government agencies (national and local), businesses, commerce and industry, policymakers and NGOs. At present, social engagement and partnerships among more than a few groups of stakeholders are rarely sustained. Silo-based approaches that can be common among government organizations—often enforced by their narrow mandates —do not allow decision-makers to explore and understand the complex interconnections that exist within urban environments. Complex trade-offs and decisions to build resilient environments should be considered, taking a long-term view of climate risks alongside disaster-risk planning and management, and ensuring that their choices will lead to economic prosperity, healthy lives and safer living environments.

Advancing scientific knowledge demands a combination of approaches towards addressing complex issues involving technical and societal concerns, including the evolution of AI and ML techniques. Integrating ideas, expertise, and practices will offer many opportunities for highly effective applications in various fields. However, our journey to advancing next-generation forecasting systems must find its way into the hands of the users in a meaningful way for these early warning communications to be effective in producing action—otherwise, all this investment will have been for nothing. This must be avoided at all costs by encouraging transdisciplinary thinking to complement the whole chain of disciplinary approaches. We believe this will enable improved co-creation and co-design of environmental outcome focused solutions for the benefit of all society.

A rethink is needed to transform current practices so that the whole of society can take appropriate action towards increased resilience. This must start with the full engagement of all stakeholders, and the reaffirmation of the critical importance of co-design and co-production of outcome-focused solutions for building resilient communities and community-based services based on pioneering transdisciplinary partnerships and living labs.

## Data Availability

This article has no additional data.

## Footnotes

^1^
FitzRoy, Robert. ‘XIV. Notice of “The Royal Charter Storm” in October 1859.’ *Proceedings of the Royal Society of London* 10 (1860): 561–567.

^2^
OCCASIONAL PAPERS ON METEOROLOGICAL HISTORY Number (rmets.org).

^3^
UK Government, 2011. Climate Resilience Infrastructure: Preparing for a Changing Climate. Secretary of State for Environment, Food and Rural Affairs, HM Government, Presentation to Parliament.

^4^
Government response to the Pitt Review—GOV.UK.

^5^
https://theconversation.com/the-climate-is-changing-so-fast-that-we-havent-seen-how-bad-extreme-weather-could-get-235726.
